# Supplementary material for: Content-rich biological network constructed by mining PubMed abstracts
Source: BMC Bioinformatics. 2004 Oct 8;5:147. doi: 10.1186/1471-2105-5-147 (PMC528731; doi:10.1186/1471-2105-5-147)
Supplement: Additional File 2 — The original results of the above study (non-essential files are deleted to keep the file size under the limit set by BMC bioinformatics). [file 1471-2105-5-147-S2.bz2 › chilibotAdditionalFile2/dip10/429ID10910894E10359/html/CCR1_CCL4.html]

 


 **CCR1** and **CCL4** 
  
Found 39 abstracts in PubMed, retrieved 10.  
 

 What does Google say? 
 PDF only 
| .edu only 

---

**Interactive relationship** (e.g. stimulation, inhibition, etc)

**Neutral relationship**- However, the remaining expression of  **CCR1**  and CCR5 correlated reciprocally with an ongoing unresponsiveness to the  **CCR1**  and CCR5 agonists CCL3 macrophage inflammatory protein 1alpha MIP 1alpha,  **CCL4**  MIP 1beta, and CCL5 regulated on activation, normal T expressed and secreted RANTES, all chemokines binding to these two receptors.  Ref: 12885942 J Leukoc Biol, 2003

**Non-interactive relationship** (e.g. studied together, co-existance, homology, etc.)

- Moreover, the forskolin mediated increases in intracellular cAMP concentrations were inhibited by the ligands for  **CCR1** , CCL3,  **CCL4** , and CCL5, suggesting that the expressed  **CCR1**  was functional.  Ref: 12651617 Am J Pathol, 2003
- After 2 to 4 weeks, L. amazonensis infected mice had significantly delayed and depressed expression of inflammatory cytokines interleukin 12 IL 12, gamma interferon, IL 1alpha, IL 1beta, CC chemokines CC chemokine ligand 3 CCL3 macrophage inflammatory protein 1alpha MIP 1alpha,  **CCL4**  MIP 1beta, CCL5 RANTES, MIP 2, and chemokine receptors  **CCR1** , CCR2, CCR5 in foot tissues and draining lymph nodes compared to the expression in L. major infected controls.  Ref: 12874303 Infect Immun, 2003
- Characterization of the chemokine receptor specificity of MIP 1 beta  [ **CCL4** ]  3 69 showed that the truncated protein not only shares the ability of intact MIP 1 beta  [ **CCL4** ]  to induce Ca 2 signaling through CCR5, but unlike the full length protein, it also triggers a Ca 2 response via  **CCR1**  and CCR2b.  Ref: 12070155 J Biol Chem, 2002
